# Enrichr in silico analysis of MS-based extracted candidate proteomic biomarkers highlights pathogenic pathways in systemic sclerosis

**DOI:** 10.1038/s41598-023-29054-5

**Published:** 2023-02-02

**Authors:** Paraskevi P. Chairta, Paschalis Nicolaou, Kyproula Christodoulou

**Affiliations:** Neurogenetics Department, The Cyprus Institute of Neurology and Genetics, 2371 Nicosia, Cyprus

**Keywords:** Computational biology and bioinformatics, Biomarkers

## Abstract

Systemic sclerosis (SSc) is a rheumatic disease characterised by vasculopathy, inflammation and fibrosis. Its aetiopathogenesis is still unknown, and the pathways/mechanisms of the disease are not clarified. This study aimed to perform in silico analysis of the already Mass Spectrometry (MS)-based discovered biomarkers of SSc to extract possible pathways/mechanisms implicated in the disease. We recorded all published candidate MS-based found biomarkers related to SSc. We then selected a number of the candidate biomarkers using specific criteria and performed pathway and cellular component analyses using Enrichr. We used PANTHER and STRING to assess the biological processes and the interactions of the recorded proteins, respectively. Pathway analysis extracted several pathways that are associated with the three different stages of SSc pathogenesis. Some of these pathways are also related to other diseases, including autoimmune diseases. We observe that these biomarkers are located in several cellular components and implicated in many biological processes. STRING analysis showed that some proteins interact, creating significant clusters, while others do not display any evidence of an interaction. All these data highlight the complexity of SSc, and further investigation of the extracted pathways/biological processes and interactions may help study the disease from a different angle.

## Introduction

Systemic sclerosis (SSc) is a chronic, heterogeneous, multisystemic, connective tissue autoimmune disease (AID). Vasculopathy, immune system abnormalities-inflammation and excessive deposition of collagen-fibrosis are characteristic features of SSc^1^. Despite intensive research, the aetiopathogenesis of the disease remains poorly understood. An interaction between genetic, epigenetic alterations and environmental factors is suggested^2,3^. Remarkably, SSc, like other AIDs, occurs more frequently in females than in males and its incidence and prevalence vary according to the geographical region and study^4^.

SSc is divided into two subgroups; limited cutaneous SSc (lcSSc) and diffused cutaneous SSc (dcSSc), based on clinical manifestations. In lcSSc, skin thickening is usually limited to the face, ankles, wrists and distal to knees and elbows. DcSSc involves skin thickening proximal to the knees and elbows and damage of internal organs (e.g. lungs and renal)^5^. Interstitial lung disease (ILD) and pulmonary arterial hypertension (PAH) are the most common types of pulmonary involvements in SSc. They are likely life-threatening as they account for 60% of death in the disease^6^.

Besides genetics and environmental factors, proteome and pathways dysregulation may play a crucial role in the pathogenesis of SSc. Proteins are considered the structural and functional molecules of the living organism and are responsible for the cell’s phenotype. Proteomics is the only way to study protein–protein interaction, the mechanism/pathways of disease, and the environmental effect at the level of the organism^7^. In addition, it is essential for biomarkers discovery; disease-specific proteins that can be used and distinguish a pathological from a non-pathological condition. These biomarkers have several applications. We can use them for better classification, early prognosis, more accurate diagnosis and therapeutic targeting of the disease. In addition, many of the candidate biomarkers might also be interconnected and help extract critical physiopathological pathways^8^.

Early-stage proteomic studies employed the two-dimensional gel electrophoresis (2DE) application. In recent years, newly developed proteomics methodologies based on MS enabled more robust studies^9^. MS, a high-throughput analytical method, allows the detection, identification and quantification of proteins in many samples such as biopsies, saliva and plasma^10^. In addition, various studies use different MS proteomic techniques such as matrix-assisted laser desorption/ionisation time-time-of-flight (MALDI-TOF) and surface-enhanced laser desorption/ionisation time-of-flight (SELDI-TOF)^8^.

In this article, we gather published candidate MS-based discovered biomarkers of SSc and then further analyse a number of these molecules selected based on specific criteria through in silico online tools for the first time to extract possible pathways involved in the development of the disease.

## Methods

### MS-based discovered biomarkers recording

All candidate MS-based proteomic biomarkers for SSc discovered until 2021 were collected/extracted through two extensive systematic reviews^8,11^. We recorded all biomarkers and grouped them based on general SSc, SSc subtypes; lcSSc and dcSSc and lung involvements (PF and ILD) related to SSc.

### Quality assessment of the MS-based studies

The quality of data reported through recorded studies was evaluated according to the eight criteria recommended by Mischak et al^12^ for biomarker identification and qualification in clinical proteomics*.* These criteria are the following: justification of the clinical question, description of the subjects, samples, methodology and statistical assessment, validation of the results, acknowledgement of limitations and authors contribution statement.

#### In silico analysis

#### Selection of proteins for in silico analysis

The selection of proteins for in silico analysis was performed using two different approaches.

The first selection of proteins for in silico analysis was performed based on three combined criteria; (1) proteins that were extracted through full text research articles, and (2) proteins that were extracted through the articles that fulfilled the first five quality assessment criteria [(1) identification and description of clinical question, (2) assessment of subjects description, (3) sampling description, (4) presentation of experimental methodology and (5) statistical approach] and (3) proteins with *p*-value ≤ 0.05 & Fold Change (FC) ≥ 1.5 or ≤ 0.67 or expressed only in disease or control samples.

The second selection of proteins for in silico analysis was performed including proteins that fulfilled the above criteria and were also validated with additional methods.

We performed in silico analysis in general SSc, dcSSc, lcSSc and lung involvements groups.

#### Enrichr

We used the Enrichr Web Server *(*https://maayanlab.cloud/Enrichr/*)*^13^ to classify the proteins/genes based on the following ontologies: (a) KEGG 2021 Human^14–16^ and (b) GO Cellular Component 2021. We performed separate pathway analyses for the upregulated and downregulated proteins.

#### PANTHER

We analysed all selected proteins using the Protein Analysis Through Evolutionary Relationships (PANTHER)—Gene List Analysis tool *(*http://www.pantherdb.org/*), Version 17.0* (released 2020–02-22)^17^. This tool classified/sub-classified proteins (and their genes) into PANTHER GO-Slim Biological Processes.

#### Venn diagram

We produced the Venn diagrams using the InteractiVenn tool *(*http://www.interactivenn.net/*)* to easily visualise common and unique pathways through different analyses and subtypes^18^.

#### STRING 11.5

We used the Search Tool for Retrieval of Interacting Genes/Proteins—STRING Version: 11.5 *(*http://string-db.org/*)*^19^ to assess the interactions of selected genes/proteins. We set the confidence level to 0.700 (high). We pulled interactions based on database, experiments, and co-expression.

## Results

### Gathering of data from previous MS-based proteomics studies

Up to date, 29 MS-based proteomics studies were performed on SSc^10,20–47^. Out of these studies, 25 were published as full text research articles. Two systematic reviews by Balanescu P et al.^8,11^ record these studies, where a total of 655 candidate proteomic biomarkers were identified (Additional file 1). Twenty-eight out of these biomarkers were further validated using additional methods.

In most studies, proteins were separated with 2DE or Two-dimensional difference gel electrophoresis (2DE DIGE) or Liquid Chromatography (LC). Proteins were identified with MALDI-TOF, SELDI-TOF or Tandem MS (MS/MS) techniques. Multiple Reaction Monitoring (MRM) and older-standard methods (IF, WB, IHC and ELISA) were used to validate these proteins.

### Quality assessment of the MS-based studies

All full text research articles included identification and description of clinical question (25/25) as well as sampling description (25/25). In the most of the studies, authors descripted the assessment of the subjects (24/25) and statistical evaluation (23/25) as well as presented the experimental methodology (24/25). However, validation of the results (14/25), acknowledge limitations (7/25) and authors contribution (10/25) are lacked from several manuscripts. All these details are shown in additional file 2.

#### In silico analysis of the 1st selection of proteins

**Figure 1:**
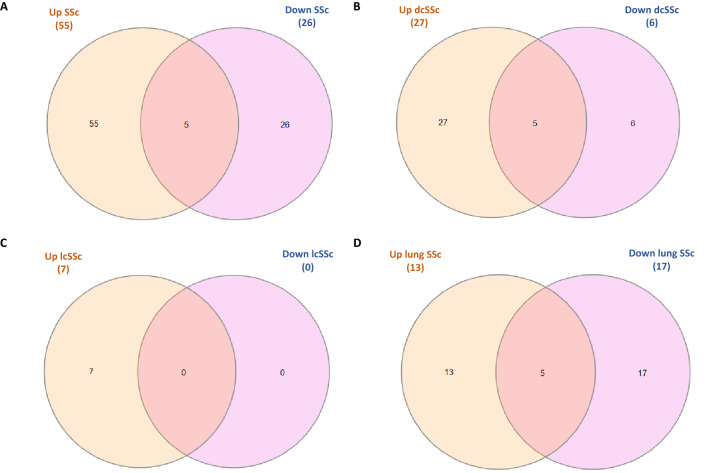
Venn diagrams indicate the number of common and unique significant pathways. These Venn diagrams indicate the number of common and unique significant pathways (*p* < 0.05) extracted through EnrichR KEGG 2021 from the significantly upregulated (*p* ≤ 0.05 & FC ≥ 1.5) and downregulated (*p* ≤ 0.05 & FC ≤ 0.67) proteins in (**A**) SSc patients, (**B**) dcSSc patients, (**C**) lcSSc patients and (**D**) lung disease related to SSc compared to controls. The overlapping areas represent the common pathways.

We performed a pathway analysis of the significantly upregulated (*p* ≤ 0.05 & FC ≥ 1.5) and downregulated (*p* ≤ 0.05 & FC ≤ 0.67) selected proteomic biomarkers. We investigated the different subtypes separately; general SSc (Additional file 3A), dcSSc (Additional file 3B), lcSSc (Additional file 3C) and lung involvements (Additional file 3D) groups. We extracted some common significant pathways from the upregulated and downregulated proteins of different groups (Fig. 1). In addition, we found a significant association of some of the extracted pathways with the three different stages of SSc pathogenesis. Among these pathways are the complement and coagulation cascades, TGF-beta signalling pathway, Antigen processing and presentation, PPAR signalling pathway, Focal adhesion, IL-17 signalling pathway, ECM-receptor interaction, Chemokine signalling pathway, Fc gamma R-mediated phagocytosis, Platelet activation, Phagosome, Oxidative phosphorylation and Leukocyte transendothelial migration.

**Figure 2:**
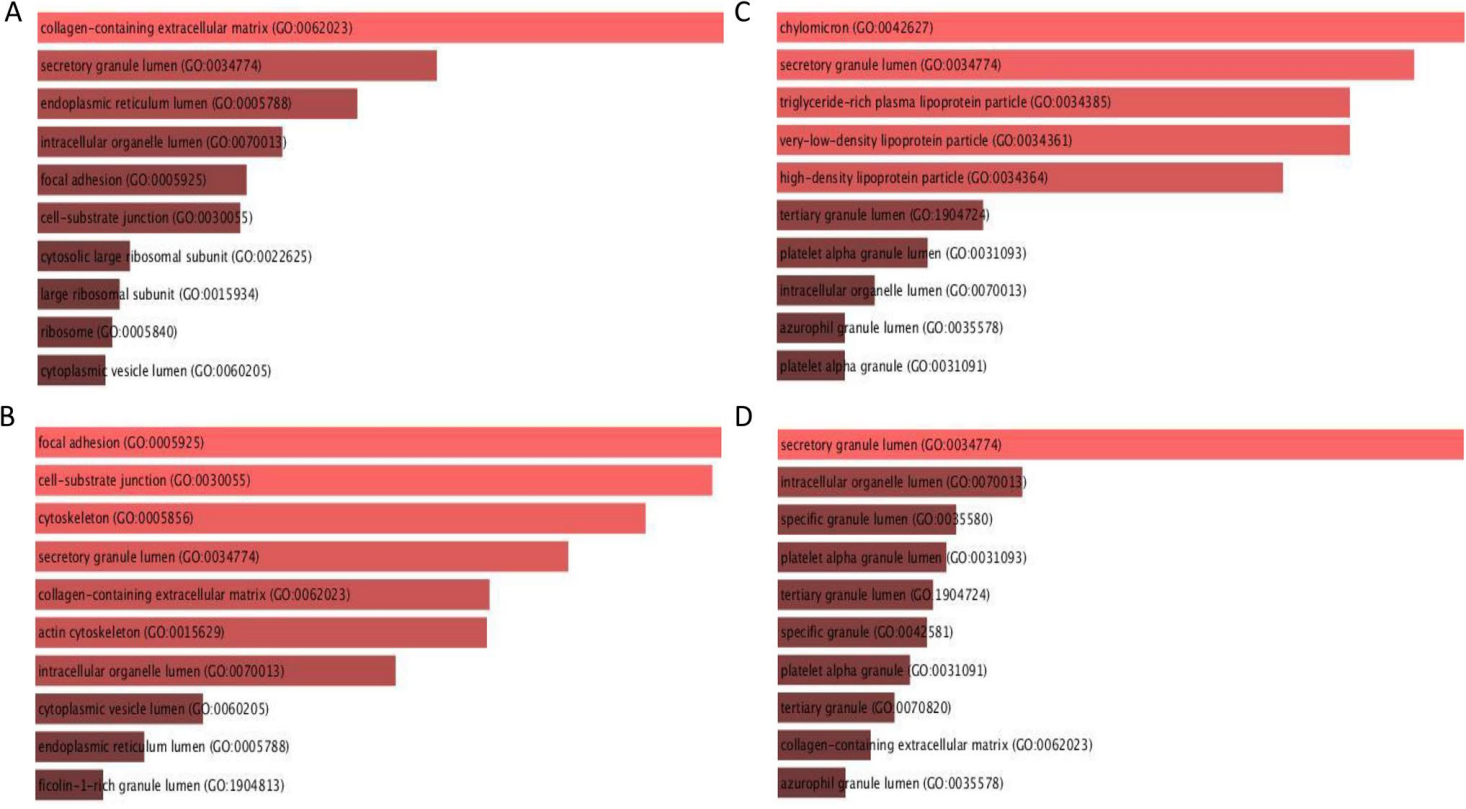
Top 10 significant Enrichr GO Cellular Component 2021. Top 10 significant Enrichr GO Cellular Component 2021 of significantly differentially expressed proteins (*p* ≤ 0.05) with FC ≥ 1.5 or ≤ 0.67 in (**A**) SSc patients, (**B**) dcSSc patients, (**C**) lcSSc patients and (**D**) lung disease related to SSc compared to controls. The brightness and the length of the bar represent the significance of the category/ term.

We used Enrichr for the GO Cellular Component 2021 analysis (Fig. 2A–D, Additional file 4). In all subgroups except lcSSc, proteins are involved in more than 100 cellular components, and more than 50 out of these components have a significant *p*-value (*p* ≤ 0.05). Collagen-containing extracellular matrix (GO:0062023) and focal adhesion (GO:0005925) are among these significant components.

**Figure 3:**
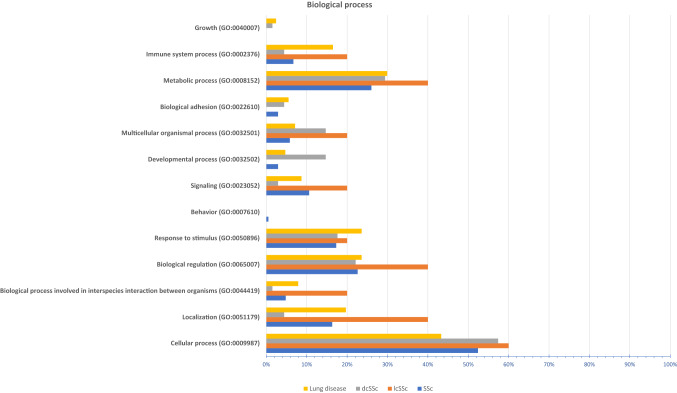
PANTHER biological process analysis. The bar chart shows the biological processes associated with the significantly dysregulated genes/proteins (*p* ≤ 0.05 & FC ≥ 1.5 or ≤ 0.67) in different subtypes; SSc, dcSSc, lcSSc and lung disease associated with SSc.

We performed PANTHER analysis to classify the proteins/genes into different biological processes (Fig. 3, Additional file 5). Dysregulated proteins of general SSc, dcSSc, lcSSc and lung involvement subgroups are implicated in 12, 12, 9 and 12 biological processes with additional sub-processes, respectively. Interestingly, 9 main biological processes are shared by all subgroups.

**Figure 4:**
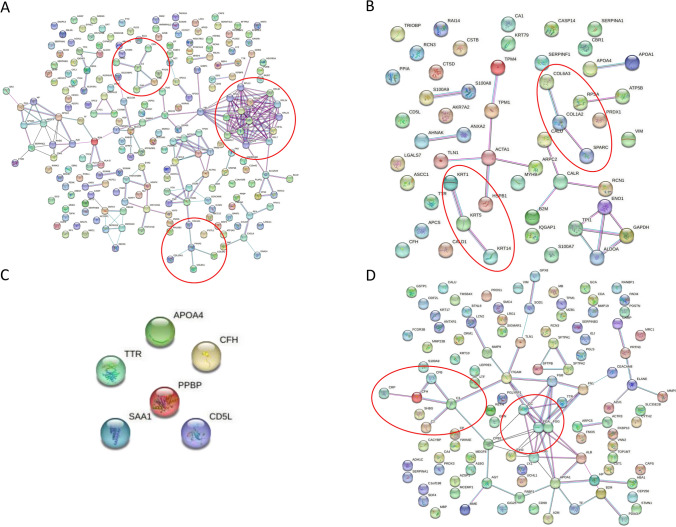
STRING (v. 11.5) analysis of the dysregulated genes/proteins. This figure reveals functional interactions between the dysregulated genes/proteins (*p* ≤ 0.05 & FC ≥ 1.5 or ≤ 0.67) in different subtypes; (**A**) SSc, (**B**) dcSSc, (**C**) lcSSc and (**D**) lung disease associated with SSc. Blue, pink and black coloured lines indicate databases, co-expression, and experimental evidence, respectively. Red cycles indicate significant clusters.

We used the STRING 11.5 database to assess whether the selected SSc candidate proteomic biomarkers have an interaction between them (Fig. 4A-D). Some clusters, as well as direct and indirect interactions, were observed in different subgroups. This analysis showed that some genes/proteins have a high confidence score of protein–protein interaction networks (e.g. PF4-CXCL8). In contrast, some others do not present any pieces of evidence for interaction (e.g. ICAM1, ABCC12) (Fig. 4A). In addition, some of the proteins interact together as they are in the same family (e.g. KRT1, KRT5 and KRT14) (Fig. 4B).

#### In silico analysis of the 2nd selection of proteins

All in silico analyses were performed on a smaller group of 21 candidate validated biomarkers for SSc. Enrichr KEGG 2021 Human, Enrichr GO Cellular Component 2021 and PANTHER biological processes analyses results are shown in Additional file 6 (A–C). STRING analysis results of these proteins are shown in Additional file 7.

## Discussion

SSc is a rheumatic AID with complex aetiopathogenesis and heterogeneous clinical manifestations. Despite that SSc is characterised by vasculopathy, immune system abnormalities and fibrosis, the molecules implicated in these processes are not fully elucidated. Several “omics” studies have been performed on SSc; however, the pathways and the mechanisms involved in the disease are still unclear^48,49^. Therefore, this study aimed to use a number of selected candidate proteomic biomarkers recorded to date for SSc to extract the central pathways implicated in the pathogenesis of SSc.

We used the Enrichr Web Server to perform pathway analysis. Several extracted pathways are associated with immunity, as immune system abnormalities and inflammation are prominent features of SSc pathogenesis. Previous studies on SSc have already reported some of the pathways and molecules we pulled through this analysis.

The complement system is the central mechanism of antibody-mediated immunity and part of the innate immune system. The primary function of this system is to recognize and eliminate foreign pathogens either through the stimulation of phagocytosis or direct killing^50^. In our study, we identified 17 dysregulated (upregulated/downregulated) molecules implicated in this pathway. Interestingly, these molecules are involved in both the coagulation and the complement (alternative, classical and lectin pathways) pathway cascades. Previous studies on SSc have also reported dysregulated proteins implicated in this pathway. Senaldi et al*.*^51^ report that C3d and ratios of C3d:C3 and Ba:factor B were higher in patients with dcSSc than controls, while C4d:C4, C4d, C3d:C3 and C3d levels were higher in patients with lcSSc compared to controls. These results show that activation of the complement system occurs in patients with SSc and might be associated with the clinical severity of the disease^51^. In a more recent study, Landi et al*.*^23^ observed decreased complement C3a levels in patients with PF compared to healthy smoking controls. In addition, pathway analysis showed that the dysregulated proteins in that study are involved in pathways associated with disease pathogenesis (alternative complement, classical complement, lectin induced complement and blood coagulation pathway)^23^. Ludwicka-Bradley et al*.*^52^ also report that imbalance of the coagulation cascade, especially release of the thrombin molecule, is associated with the earliest SSc events following tissue injury and is mainly observed in SSc patients with lung disease.

Antigen processing and presentation is an additional pathway extracted through our analysis (7 molecules) associated with SSc and all AIDs. This pathway is responsible for processing and presenting the foreign antigen to the appropriate immune cells through the Major Histocompatibility Complex (MHC) mechanism^53^. Acosta-Herrera et al*.*^54^ have reported the association of this pathway with SSc. They performed sequential conditional analyses of the human leukocyte antigen (HLA) region. They conclude on seven amino acids, nine classical alleles and nine Single Nucleotide Polymorphisms (SNPs) associated with the disease and its subtypes.

Chemokine signalling and IL-17 signalling are two additional pathways extracted from our analysis and associated with immune system regulation. The chemokine signalling pathway was extracted with a significant *p*-value only from the general SSc downregulated proteins (4 molecules). Chemokines, also known as chemotactic cytokines, regulate the recruitment and migration of immune cells. These molecules were found to be associated with several rheumatic diseases, including SSc, systemic lupus erythematosus (SLE) and rheumatoid arthritis (RA)^55^. For instance, Van Bon et al*.*^28^ reported that the plasma level of CXCL8 is higher in SSc patients than in healthy controls, while PPBP is lower. However, Scambi et al*.*^26^ reported that the levels of PPBP is higher in serum of dcSSc and lcSSc patients compared to healthy controls.

IL-17 family is a group of six inflammatory cytokines that are associated with cancer progression and AIDs. Despite that IL-17 signalling pathway is a cytokines pathway that includes several activators and inhibitors, previous studies reported that it might be implicated in fibrosis of SSc pathogenesis^56^. However, its exact role on the disease has not been elucidated. Some studies reported that IL-17A expression is higher in serum of SSc patients, and others reported it lower in SSc patients than in healthy controls^56^. In addition, Wakhlu et al*.*^57^ showed that high levels of IL-6, IL-17A and TGF β1 molecules in serum of SSc patients are associated with skin fibrosis and ILD. This pathway was significantly extracted from three out of four subgroups in our analysis, but 7 different molecules than the above are implicated. Although many studies reported dysregulated chemokine and interleukin molecules associated with SSc, chemokine and IL-17 signalling pathways are extracted through pathway analysis using SSc dysregulated proteins for the first time in our study.

Leukocyte transendothelial migration is also an essential pathway. CD4 + T lymphocytes transendothelial migration is enhanced in SSc, and the migrating cells present an activated phase^58^. In a previous study, Xu et al*.*^59^ performed KEGG pathway analysis of ITGA5, ITGB2, and ITGB5 that were found to be upregulated in the skin of SSc patients compared to control and reported that these proteins are implicated in the leukocyte transendothelial migration pathway. In our study, this pathway was significantly extracted from general SSc (upregulated proteins) and lung disease related to SSc (downregulated proteins) subgroups (ITGB1;ACTN1;ICAM1;ITGAM;MMP9). Our results show that ITGAM and MMP9 proteins are mainly dysregulated for leukocyte transendothelial migration, indicating that they might promote lung disease related to SSc by affecting this procedure.

Interestingly, phagosome, as well as Fc gamma R-mediated phagocytosis pathways, were also extracted in our analysis. Phagocytosis is a fundamental process for innate immune response where the specialised phagocytes such as macrophages, monocytes, dendritic cells, and neutrophils recognise, engulf and eliminate the pathogenic microorganisms^60^. This process has a crucial role in maintaining tissue homeostasis by removing apoptotic cells^61^. Receptors of phagocytes are categorized into opsonic and non-opsonic. The non-opsonic receptor directly recognises substances on the phagocytic target surface, while the opsonic receptor identifies host-derived proteins bound to the foreign particle. Complement and Fc are the most essential and well-characterised opsonic phagocytic receptors^61^. Previous studies showed that defects in the clearance of apoptotic cells might lead to several diseases, including AIDs^62^. Davis et al*.*^63^ showed that anti-FcγR autoantibodies are detected in the serum of SSc patients but not in controls. In addition, Kadono et al*.*^64^ in a more recent study, report that the levels of anti-FcγRIIB/C autoantibodies in the serum of SSc patients are significantly higher than in healthy controls. These findings support the idea that phagocytosis is implicated in the pathogenesis of SSc, but further studies are needed to elucidate its exact role.

Peroxisomes are essential organelles that regulate lipid homeostasis. They also interact with some pathogens to escape the immune response, turn off/on pivotal cellular immune signalling and control the interaction between host and pathogen^65^. Peroxisome proliferator-activated receptors (PPARs); PPAR-α, -β/δ, and γ are nuclear receptors that regulate several processes, including inflammation, differentiation and cell proliferation^66^. Previous studies prove that PPAR-γ is implicated in the pathogenesis of SSc, especially aberrant expression of PPAR-γ contributes to fibrosis and vascular remodelling stages^67^. Shiwen et al*.*^68^ showed reduced expression of PPAR-γ in fibroblasts of patients with dcSSc compared to healthy controls. In addition, Marangoni et al*.*^69^ reported that PPAR-γ is also related with SSc at the genetic level, as PPAR-γ intronic SNP (rs10865710) is associated with SSc susceptibility. Interestingly, in our study, PPARs signalling pathway was extracted, but the implicated proteins are included in all three signals and not only in the PPAR-γ part.

TGF-beta is a pathway interconnected with PPAR signalling and significantly extracted from our analysis (3 molecules in general SSc analysis). Wei et al*.*^70^ suggested that PPAR-γ expression in SSc is suppressed by excessive activity of TGF-ß and may contribute to the abnormal activation of fibroblast and fibrogenesis. This signalling pathway regulates several cellular processes, including extracellular matrix (ECM) synthesis, cell differentiation, apoptosis and cell growth^71^. Increased levels of TGF-β1 in tissue and serum of patients with SSc suggest that it might be implicated in fibrosis. In addition, studies showed that TGF-β signalling could be a mediator of experimental PAH and blocking of this signalling was shown to reduce the severity of PAH^71^. Dantas et al*.* assessed the levels of the active TGF-β1 isoform in skin biopsies and serum from patients with SSc and healthy controls. They reported that the serum level of TGF-β1 was significantly higher in patients compared to controls. At the same time, no significant difference was observed in the mRNA expression of TGFB1 in skin biopsies of patients with SSc compared to controls. They also noted that patients with SSc who have high levels of TGF-β1 in serum are more likely to have dcSSc, DU, SSc (Anti-Topoisomerase) ATA + , higher Rodnan skin score and lung fibrosis. These results suggest that this molecule might be a marker for vascular and fibrotic manifestations of SSc^72^.

The ECM-receptor interaction is also included in the essential signalling pathways associated with SSc, especially in fibrosis. Several previous studies identified dysregulated proteins implicated in this pathway and suggest that it plays a crucial role in developing the disease^59,73^. Xu et al*.*^59^ showed that protein and mRNA levels of three integrins (ITGB5, ITGB2 and ITGA5) are upregulated in SSc patients than in controls. Pathway analysis showed that these proteins/genes are implicated in leukocyte transendothelial migration, focal adhesion, ECM–receptor interaction and ECM turnover. This pathway was extracted in our study, not only from integrin proteins but also from the collagen family proteins. Focal adhesion and cell adhesion molecules pathways were also extracted from our analysis. Adhesion molecules are proteins that mediate cell-to-ECM as well as cell-to-cell interaction. Based on their function, these molecules are located on the surface of different cells and implicated in several processes, including T-Cell Mediated Immunity and leukocyte migration and fibrogenesis^74,75^. Although these two pathways were not discussed in depth in previous studies, Hasegawa et al*.*^76^ report that serum adhesion molecules can be used as predictive biomarkers for early SSc. In addition, in our previous study, we report that the ICAM1 protein is significantly over-expressed in affected than unaffected skin biopsies of patients with SSc^10^.

Platelet activation and oxidative phosphorylation are also crucial pathways that contribute to all stages of SSc pathogenesis: vasculopathy, autoimmunity and fibrosis and they were extracted from our analysis^77–79^. Agache et al*.*^80^ reported that platelet activation molecules such as P-selectin are associated with disease severity and activity. It was also reported that platelets of SSc patients have a high response to collagen, adenosine diphosphate, adrenaline and 5-hydroxytryptamine, which are correlated with different processes^77^. Even though oxidative phosphorylation is essential for energy production in the organism, an abnormal function of this mechanism might lead to a pathological condition through the overproduction of Reactive Oxygen Species (ROS). For example, high levels of ROS were observed in the skin fibroblast of patients with SSc compared to healthy controls^79^. In addition, oxidative stress, which is the imbalance and accumulation of ROS, may cause endothelial cells activation and damage/dysfunction^78^. All of these pathways discussed above were extracted with a significant *p*-value either in one or more of our 1st selection analyses and might be implicated in the pathogenesis of SSc. Although in the 2nd selection the number of validated proteins was relatively small, complement and coagulation cascades and IL-17 signalling pathway were also extracted through these analyses with a significant *p*-value. Interestingly, Wnt signalling pathway, that is associated with SSc, was extracted through the 2nd selection analyses only with a significant *p*-value (Additional file 6A). Wnt signalling is an essential pathway that is implicated in several processes such as organogenesis, cell migration and cell fate during the embryonic development^81^. Wei et al*.*^82^ used genome-wide expression datasets and reported a decreased expression of Wif1 and Dkk2 (WNT antagonists) and an increased expression of Lef1 (Wnt target) and Frizzled2 (Wnt receptor) in dcSSc compared to healthy controls skin biopsies. In addition, an immunohistochemical study showed an elevated expression of nuclear β- catenin (transcriptional coactivator) in these skin biopsies. These evidences suggest a hyperactivation of Wnt signalling in patients with SSc and support that this pathway plays a key role in the pathogenesis of fibrosis in SSc^82^.

Enrichr analyses were also performed to classify the proteins based on their cellular components. These analyses showed that the recorded SSc associated proteins are located in many cellular components, some of them in more than one component (Additional file 4). The variety of cellular components confirms the complexity and heterogeneity of the disease. In addition, nine cellular components were extracted with significant *p*-value and were common in all analyses; general SSc, dcSSc, lcSSc and lung disease related-SSc. In all analyses except from lcSSc, the collagen-containing extracellular matrix (GO:0062023), related to SSc fibrosis, was extracted in the top-10 cellular components. Interestingly, collagen-containing extracellular matrix (GO:0062023) was also extracted with a significant *p*-value in the general SSc group of the 2nd selection analysis (Additional file 6B).

Biological process analysis is carried out to show the specific objective that an organism is programmed to achieve. Our 1st analysis of altered proteins indicated that these proteins are involved in 13 different biological processes and many sub-processes (Additional file 5). The percentage of genes classified in each biological process is varied based on the SSc subgroup. Cellular homeostasis that was extracted from our analysis was also extracted from DAVID in a previous study by Corallo et al.^36^. However, our analysis is more enriched as different databases give a different outcome. Biological process analysis of the 2nd selection of validated proteins showed that they are involved in 11 different biological processes and some sub-processes (Additional file 6C).

STRING analysis was performed to assess whether the recorded proteins are interconnected and create clusters. Direct and indirect interactions via databases, experiments and co-expression with a high confidence score (0.700) were extracted and are presented in Fig. 4A-D. Different interactions and clusters are observed in different subgroups. For example, in the analysis of general SSc (Fig. 4A), the cluster with the highest score contains several ribosomal proteins. Clusters that contain the complement system and collagen proteins were also observed. In the analysis of dcSSc (Fig. 4B), the collagen and keratin protein clusters associated with fibrosis were extracted. In addition, analysis of lung disease associated with SSc extracted the complement system and fibrinogen proteins cluster, related with the pathogenesis of SSc. Although some proteins show interactions between them with a high score, many do not display any pieces of evidence of an interaction. However, STRING analysis of the 2nd selection of proteins does not show any essential interaction between them (Additional file 7).

## Conclusions

This study shows that many proteins with different functions are implicated in the pathogenesis of SSc. Pathway analysis confirms the heterogeneity of the disease as the recorded dysregulated proteins are involved in approximately 240 different pathways. Different pathways and molecules are involved in various stages of SSc pathogenesis and different SSc subtypes. Some of the extracted pathways are also related to other diseases, including AIDs, indicating that they share common mechanisms. Some of the significantly extracted pathways were associated with SSc in previous studies, but some of them were not studied in depth. Cellular component and biological process analyses confirmed the complexity of the disease as the recorded molecules are implicated in 13 different biological processes and located in more than 230 cellular components. All these analyses that were performed for the first time for all the MS-based SSc proteomic biomarkers in our study are essential for producing new data associated with the disease pathogenesis, for the identification/determination of the biological mechanisms and molecular interactions that lead to the disease, and for developing novel aspects for further studies.

## Supplementary Information

Supplementary Information 1.

Supplementary Information 2.

Supplementary Information 3.

Supplementary Information 4.

Supplementary Information 5.

Supplementary Information 6.

Supplementary Information 7.

## Data Availability

The datasets generated and/or analysed during the current study are available through Balanescu P et al*.*^8,11^. This data can be found in Additional file 1.

## Abbreviations

AID: Autoimmune disease
dcSSc: Diffused cutaneous SSc
ECM: Extracellular matrix
FC: Fold change
HLA: Human leukocyte antigen
ILD: Interstitial lung disease
lcSSc: Limited cutaneous SSc
LC: Liquid chromatography
MHC: Major histocompatibility complex
MS: Mass spectrometry
MALDI-TOF: Matrix-assisted laser desorption/ionisation time-time-of-flight
MRM: Multiple reaction monitoring
PPARs: Peroxisome proliferator-activated receptors
PANTHER: Protein analysis through evolutionary relationships
PAH: Pulmonary arterial hypertension
ROS: Reactive oxygen species
RA: Rheumatoid arthritis
SNPs: Single nucleotide polymorphisms
SELDI-TOF: Surface-enhanced laser desorption/ionisation time-of-flight
SLE: Systemic lupus erythematosus
SSc: Systemic sclerosis
MS/MS: Tandem MS
2DE DIGE: Two-dimensional difference gel electrophoresis
2DE: Two-dimensional gel electrophoresis
UPS: Ubiquitin–proteasome system
