# Supplementary figures and images for: Enrichr in silico analysis of MS-based extracted candidate proteomic biomarkers highlights pathogenic pathways in systemic sclerosis
Source: Sci Rep. 2023 Feb 2;13:1934. doi: 10.1038/s41598-023-29054-5 (PMC9894849; doi:10.1038/s41598-023-29054-5)

## Slide 1
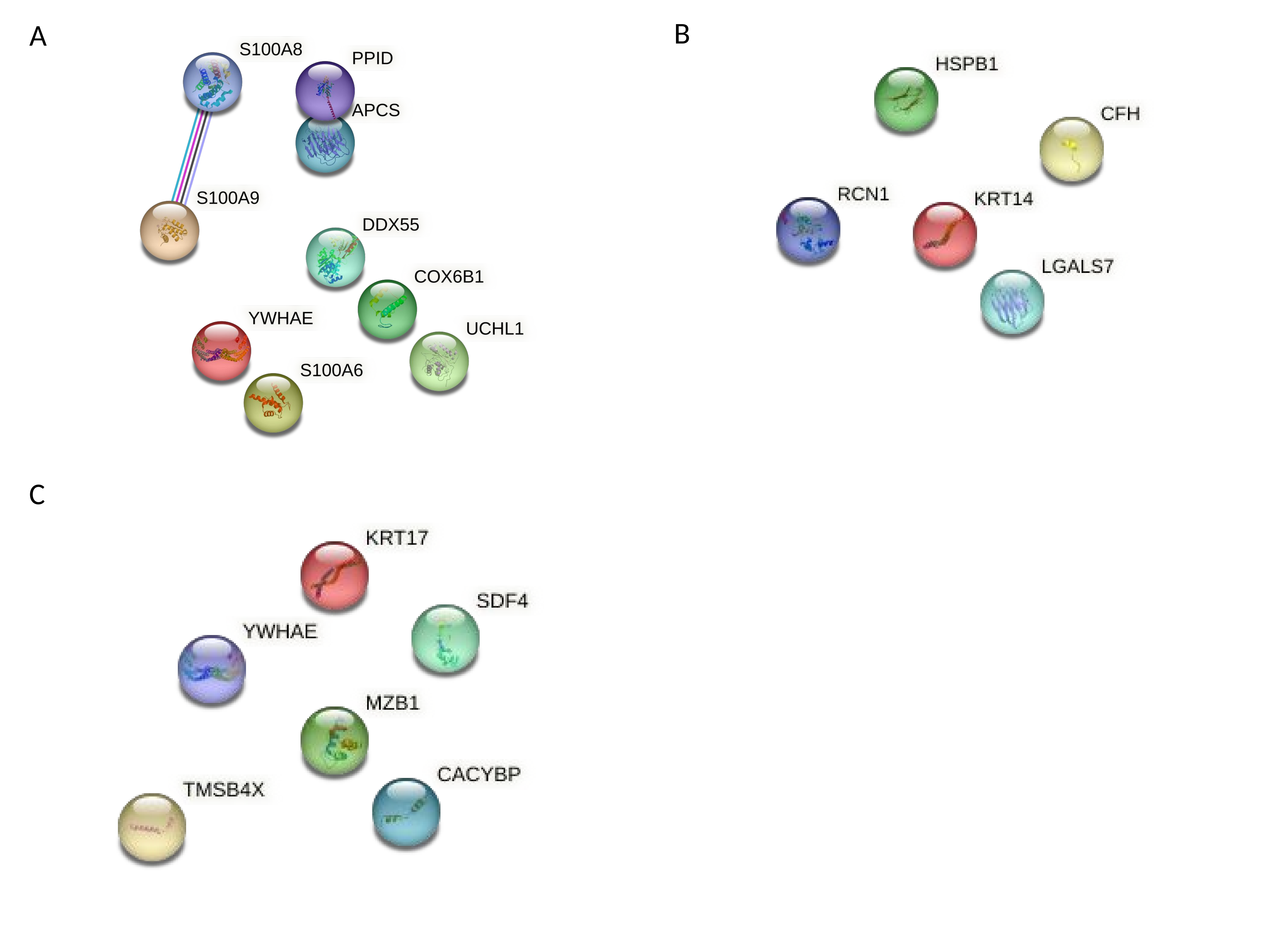

B
A
C

Supplement: Supplementary file 7 — Supplementary Information 7. [file 41598_2023_29054_MOESM7_ESM.pptx]
